# Impact of Ringer’s Solution Challenge Stress to Immunostimulatory Experiment, Insights From Japanese Flounder

**DOI:** 10.3389/fphys.2020.612036

**Published:** 2020-12-04

**Authors:** Jinxiang Liu, Zan Li, Yujue Wang, Quanqi Zhang

**Affiliations:** ^1^Key Laboratory of Marine Genetics and Breeding, Ministry of Education, Ocean University of China, Qingdao, China; ^2^Laboratory for Marine Fisheries Science and Food Production Processes, Qingdao National Laboratory for Marine Science and Technology, Qingdao, China; ^3^Laboratory of Tropical Marine Germplasm Resources and Breeding Engineering, Sanya Oceanographic Institution, Ocean University of China, Sanya, China

**Keywords:** Ringer’s, stress, RNA-seq, immune response, glycolysis

## Abstract

Ringer’s or phosphate buffer saline (PBS) solution buffer usually was used as dilution butter in intraperitoneal injection. Stress could activate immune response, inflammatory response and glycogen metabolic process. The impact of solution buffer as a stressor to immune system was ignored in immunostimulatory experiment. In this report, we tested the hypothesis that the innate immune response and glycogen metabolic process were altered when it were challenged with Ringer’s in Japanese flounder (*Paralichthys olivaceus*). RNA-seq was performed after challenge with Ringer’s at 8 h and 48 h. The data revealed that the expression profiles of blood, gill, and kidney were significantly changed. Differentially expressed genes (DEGs) were identified, and energy metabolic and immune-related genes were up-regulated or down-regulated obviously. GO and KEGG analyses showed that DEGs were mainly enriched in innate immune terms and pathways. Weighted gene co-expression networks analysis (WGCNA) also indicated the highest association module with stress. A total of 16 genes were detected in the gray module, which were immune-related and metabolic-related genes. These results provided fundamental information on intraperitoneal injection with solution buffer. It offered useful clues to further explore the functional mechanism of stress and immunity.

## Introduction

Stress is a general term applying to a situation in which organism is subjected to a challenge (Zhang et al., 2012). The stressors induced stress response can be mainly classified into different types. Firstly, physical and environmental stressors, including injuries, manipulations, handing, water temperature, dissolved oxygen, nitrogen compounds, salinity, pH, contaminants, and pathogens. Secondly, social and symbolic stressors, such as dominance, crowding, aggressiveness, and threat. Thirdly, acute and chronic stressors, for instance, predation, and fighting (Tort, 2011). It was demonstrated that stress could suppress or enhance the pathways involved in immune response (Dhabhar et al., 1995; Dhabhar, 2002). Stress also caused a wide range of physiological mechanisms except for immune, including the changes of gene and protein, metabolism, energetics, endocrine, and neural (Tort, 2011). In previous studies, it was reported that cytokines and neuropeptides performed roles in both neuroendocrine and immune systems after challenged with stress in fish (Ottaviani and Franceschi, 1996). The innate immunity and inflammatory response could be activated or enhanced after stress. It was investigated that the lysozyme and C3 was increased after acute stress in rainbow trout and sea bream (Oriol Sunyer and Tort, 1995; Demers and Bayne, 1997).

The mechanism of these interactions has been mostly studied in higher vertebrates, but less information were available in fish. The studies focused on the regulation stimulated by high or low temperature. The normal expression profiles of kidney and gill were changed after challenged with high temperature in rainbow trout and Yesso scallop, and immune-related genes were up- and down-regulated (Huang et al., 2018; Jiang et al., 2018). A series of evidences indicated the immune response stimulated by stress mediated by cortisol and glucocorticoids. Immune suppression was correlated with high level of cortisol in sea bream and red porgy (Tort et al., 1998; Montero et al., 1999; MacKenzie et al., 2006; Rotllant and Tort, 2010). In a recent research, obvious oxidative burst was found in juvenile Chinook salmon leukocytes, and followed the cortisol concentration significantly increased after stress treatment (Herron et al., 2018). Glucocorticoid could also increase leukocytes of coho salmon after stress (Maule and Schreck, 1991).

Ringer’s or PBS was usually used for immunostimulatory experiment as solution buffer to dilute bacteria. In fact, Ringer’s challenge was a stress, which could induce immune response. We should recognized it correctly, which might produce noise and interfere the result during immunostimulatory experiment. It could be recognized correctly, and exclude the noise caused by stress during immunostimulatory experiment. In our study, RNA-seq was used the estimate the influence of Ringer’s challenge at 8 h and 48 h post injection. Immune-related tissues were selected to detect the expression change after injection at transcriptome level. We demonstrated that the genes related to inflammatory response, innate immune, and metabolic process changed obviously. The results could provide a theoretical basis for the immunostimulatory experiment, especially intraperitoneal injection. The impact of solution buffer should not be ignored. It could be a reference for researchers to choose the better experimental method among injection, soaking, and feeding.

## Materials and Methods

### Ethics Statement

All the experiments were conducted according to the Guidelines for the Institutional Animal Care and Use Committee of the Ocean University of China.

### Fish and Sample Collection

The Japanese flounders used for the experiment were collected by the Yellow Sea Aquatic Product Co., Ltd., Shandong. A total of 12 healthy 1-year-old individuals were randomly selected for challenge experiment. The fish were divided into two groups denoted as the blank control group (BC group) and treatment group, which included four and eight individuals, respectively. During the experiment, the treatment group received an intraperitoneal injection of 1 mL sterilized Ringer’s solution buffer, and the BC group did not receive any treatment. The fish were maintained in sterilized seawater with sufficient oxygen after challenge. Before sampling, fish were anesthetized with MS-222. Four individuals were randomly collected from the BC group at 0 h post injection. At 8 h and 48 h post injection, four individuals were randomly collected from the treatment group. Tissues from blood, gill and kidney were collected and snap-frozen in liquid nitrogen and stored at −80°C. After collection, the samples were tested to exclude the infection of pathogen (Li et al., 2018).

### RNA Isolation, cDNA Library Construction and Illumina Sequencing

Total RNA was extracted using TRIzol Reagent (Invitrogen, Carlsbad, CA, United States) in accordance with the manufacturer’s protocol, treated with RNase-free DNase I (TaKaRa, Dalian, China) to degrade genomic DNA, and then frozen at −80°C. RNAClean RNA Kit was applied to remove proteins. The quality and quantity were evaluated via 1.5% agarose gel electrophoresis and spectrophotometry using NanoPhotometer Pearl (Implen GmbH, Munich, Germany) and Agilent 2100 Bioanalyzer (Agilent Technologies, Santa Clara, CA, United States). The RNA-seq libraries of different tissues and time points were constructed by using Illumina TruSeq RNA Sample Prep Kit (Illumina, San Diego, CA, United States) in accordance with the manufacturer’s instruction. A total of 18 libraries were constructed, including BL-BC-1/2, BL-8 h-1/2, BL-48 h-1/2, G-BC-1/2, G-8 h-1/2, G-48 h-1/2, K-BC-1/2, K-8 h-1/2, and K-48 h-1/2. During the library construction, two biological replicates of each sample were used. Equal molar ratios of RNA from any two of four individuals were pooled as one replication at each time point in the same tissue. The remaining two individuals were pooled as another replication. Then the libraries were subjected to paired-end sequencing of 150 bp on the Illumina HiSeq 4000.

### Data Processing and Bioinformatics Analysis

Raw reads were cleaned by removing adaptors and low quality sequences using FastQC. TopHat was used to map the reads to the reference genome. Default values were set for the parameters of TopHat read mapping (Kim and Salzberg, 2011). Then the mapping files were analyzed using Cufflinks to assemble the reads into transcripts for each dataset (Roberts et al., 2011). Complete transcripts were obtained by merging the assemblies of all datasets using Cuffmerge. Gene expression levels were measured by Fragments Per Kilobase Million (FPKM) (Trapnell et al., 2012). The expressed genes were annotated by NR, Swiss-Prot, GO, and KOG databases (Kanehisa et al., 2008).

### DEGs Identification, GO and KEGG Enrichment

Differentially expressed genes among BC groups and treatment groups at different time point and different tissues were detected by FPKM. The FPKM was calculated by Cuffdiff. Genes with an adjusted log2FoldChange ≥ 1 or log2FoldChange ≤ −1, and *P* < 0.01 were considered as DEGs. The DEGs were enriched by GO terms and KEGG categories using DAVID (Huang et al., 2008). The visualization of global similarities and differences were accomplished by MA plot, and R packages were used for these analysis. The significance of GO and KEGG enrichment analysis was determined via modified Fisher’s exact test (EASE ≤ 0.05).

### Construction of Weighted Gene Co-expression Network

Gene co-expression networks were constructed using the WGCNA approach with R packages. Genes expressed in different tissues and time points were retained for co-expression network construction by WGCNA (Langfelder and Horvath, 2008). The co-expression adjacency matrix were formed based on correlation between each gene (genes FPKM < 0.5 were deleted). Cluster analysis was performed with flashClust function from the package flashClust, and modules were identified with the cutreeHybrid function of the package Dynamic Tree Cut. The calculation of module eigengene (ME) were carried out by the MEs function of WGCNA package. Gene modules with common expression patterns associated with particular trait were detected based on the correlation between ME and trait.

### qRT-PCR Validation

A total of nine DEGs were selected for qRT-PCR validation. Specific primer pairs were designed by IDT (Supplementary Table 1). qRT-PCR was performed using the SYBR Premix Ex TaqII on LightCycler 480. *β-actin* was selected as reference gene. Amplification conditions were as follows: 95°C for 60 s and then 45 cycles of 95°C for 5 s, 60°C for 30 s for 45 s. The relative quantities were calculated using the 2^–ΔΔCt^ comparative Ct method. The Pearson correlation coefficient analysis was performed between qRT-PCR assay and RNA-seq data.

## Results and Discussion

### Illumina Sequencing and Reads Mapping

A total of 18 libraries were sequenced, and 740,615,986 raw reads were acquired. Table 1 shown the clean reads, valid ratio and mapped reads ratio of each library after quality control. The paired-end reads were mapped to the reference genome, and more than 85% of them were mapped to the *P. olivaceus* genome. Meanwhile, more than 15,000 genes were annotated in each sample.

**TABLE 1:** Summary statistics of transcriptome sequencing data.

Sample	Raw reads	Clean reads	Q30	Mapped reads ratio (%)
BL-BC-1	39386050	38249960	96.61	85.4
BL-BC-2	37893132	36471298	96.25	86.2
BL-8h-1	38968468	37891324	96.87	84.8
BL-8h-2	43018882	42167744	96.53	85.6
BL-48h-1	43378088	41847770	96.93	86.7
BL-48h-2	34815162	33693164	96.66	85.1
G-BC-1	46623232	44808810	96.09	84.0
G-BC-2	35757094	34188358	96.36	83.1
G-8h-1	37358864	36658130	96.54	84.2
G-8h-2	44639998	43288968	96.42	83.5
G-48h-1	50302230	49194286	96.33	81.7
G-48h-2	46566514	44290268	96.34	83.2
K-BC-1	34654974	33660130	96.54	81.8
K-BC-2	43336872	41552026	96.49	80.6
K-8h-1	48870804	47839838	96.71	80.0
K-8h-2	41977128	39794424	96.37	82.0
K-48h-1	32195342	31520502	96.38	85.1
K-48h-2	40873152	39124050	96.33	80.3

### DEGs Identification Between Control and Treatment Groups

**FIGURE 1:**
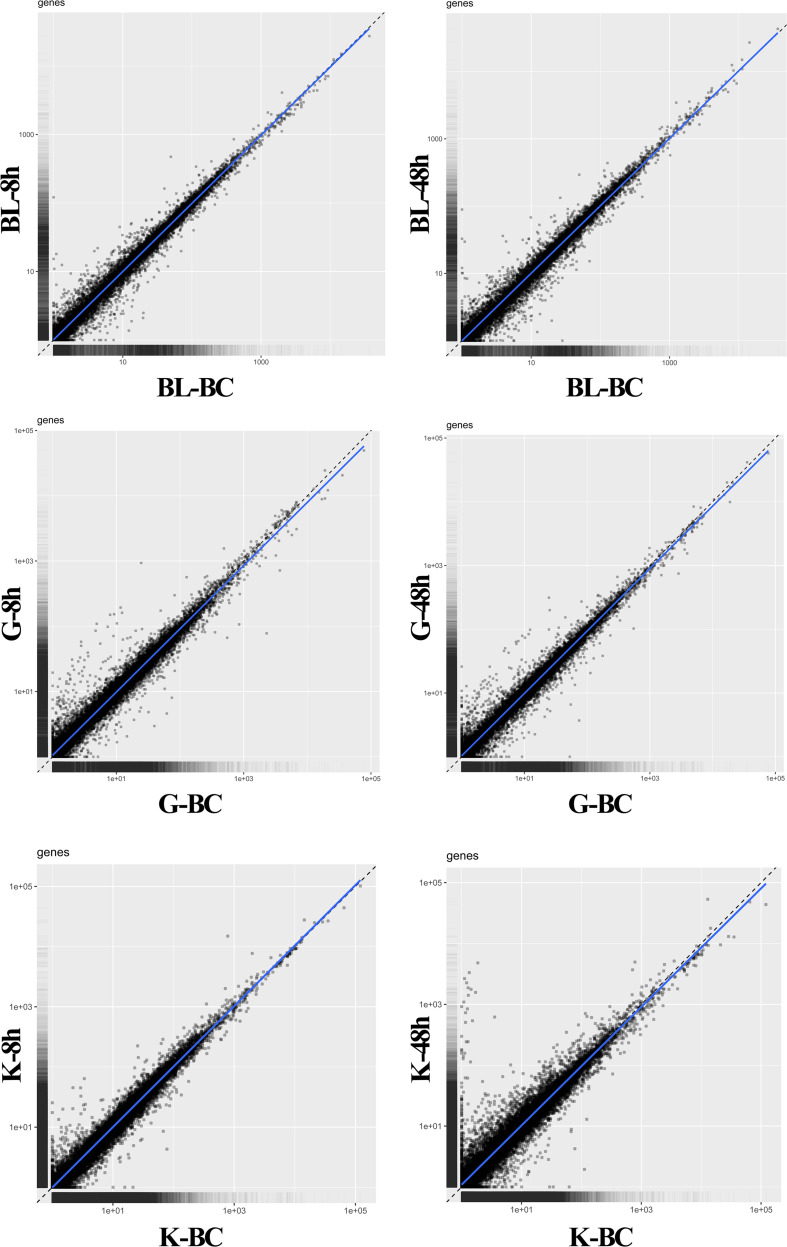
MA plot analysis between BC group vs. 8 h and BC group vs. 48 h post injection in blood, gill, and kidney. BC, blank control; BL, blood; G, gill; K, kidney.

**FIGURE 2:**
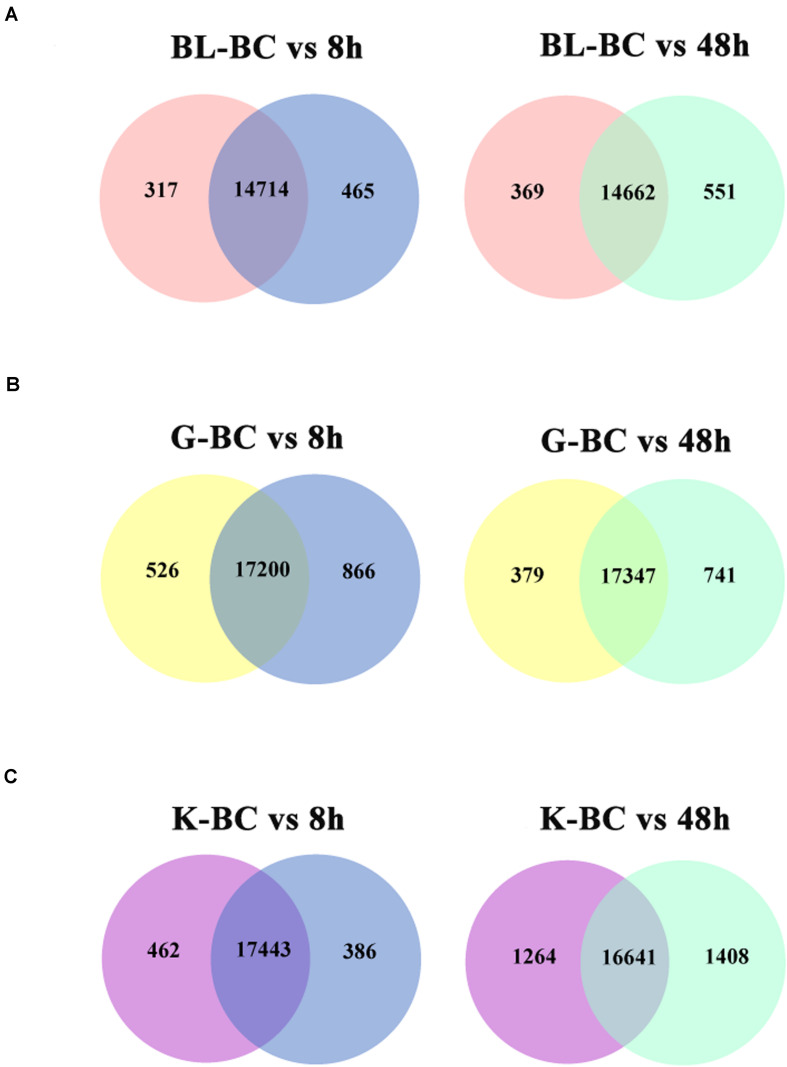
Venn diagram shown the numbers of expressed genes and DEGs in different tissues after challenged with Ringer’s. **(A)** DEGs in blood. **(B)** DEGs in gill. **(C)** DEGs in kidney.

MA plot analysis was conducted between different time points and BC groups in blood, gill and kidney. It was shown that most of genes demonstrated distributed in diagonal, and only parts of them deviated (Figure 1). DEGs were identified after Ringer’s challenge. In blood, 317 DEGs were found at 8 h post injection, which contained 184 genes were up-regulated and 133 genes were down-regulated. 369 DEGs were detected at 48 h post injection, including 152 up-regulated genes and 217 down-regulated genes (Figure 2A and Supplementary Table 2). In gill, 526 and 379 DEGs were found at 8 h and 48 h post injection, respectively. Specifically, 322 genes were up-regulated and 204 genes were down-regulated at 8 h post injection. 198 genes were up-regulated and 181 genes were down-regulated at 48 h post injection (Figure 2B and Supplementary Table 3). In kidney, a total of 462 and 1264 DEGs were identified at 8 h and 48 h post injection compared with BC group. 192 genes were up-regulated and 270 genes were down-regulated at 8 h post injection. 839 genes were up-regulated and 425 genes were down-regulated at 48 h post injection (Figure 2C and Supplementary Table 4). The results indicated that the expression profile had been changed after challenge with Ringer’s solution buffer. Usually, Ringer’s or PBS were used for diluting bacterial fluid during intraperitoneal injection and immunostimulatory experiment. It could be a kind of stress when Ringer’s or PBS buffer was injected as control group. Stress might cause a series of immune response (Dhabhar et al., 1995; Dhabhar, 2002), and generate noise during the experiment. It was important to choose the better method in challenge experiment.

### GO and KEGG Functional Analysis of DEGs

**FIGURE 3:**
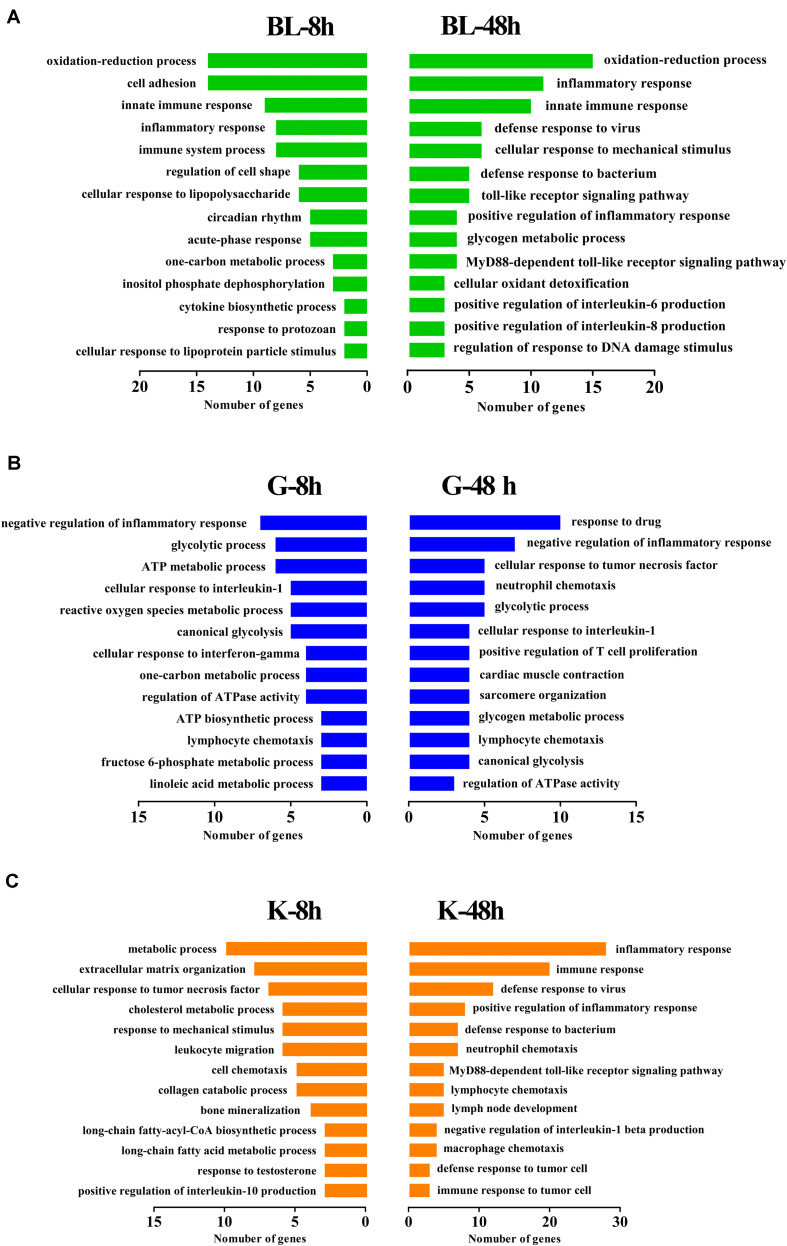
GO enrichment analysis of DEGs in different tissues. DEGs were annotated to different GO terms. **(A)** GO enrichment of DEGs in blood at 8 h and 48 h post injection. **(B)** GO enrichment of DEGs in gill at 8 h and 48 h post injection. **(C)** GO enrichment of DEGs in kidney at 8 h and 48 h post injection.

Gene ontology (GO) and Kyoto encyclopedia of genes and genomes (KEGG) analyses were performed on DEGs at different time points in different tissues. The DEGs were significantly enriched in several GO terms in biological process, cellular component and molecular function levels. In blood, the terms involved in innate immune response, inflammatory response and immune system process were enrich significantly from 317 DEGs at 8 h post injection. Meanwhile, the terms related to immune response were enriched from 369 DEGs at 48 h post injection, including inflammatory response, innate immune response, defensing response to virus and TLR signaling pathway (Figure 3A and Table 2). In gill, the terms about inflammatory response, glycolytic metabolism and ATP metabolic process were detected from DEGs after 8 h and 48 h challenge (Figure 3B and Table 3). In kidney, it was found that there were some terms related to metabolic process, response to mechanical stimulus and immune response at 8 h post injection. Besides, an amount of genes were enriched to the terms about inflammatory response, immune response, and signaling pathways about immune response (Figure 3C and Table 4). Basing on the results of GO enrichment, it was investigated that the stimulation of Ringer’s challenge changed the expression of genes involved in inflammatory. In addition, the genes related to glycolytic metabolism were also changed. Our results was consisted with the previous studies on stress, the immune-related genes and glycolytic metabolism-related genes were changed in other stresses. Meanwhile, inflammatory factors even were up-regulated (Calcagni and Elenkov, 2006; Rogatsky and Ivashkiv, 2006; Borghetti et al., 2009). It was a kind of stress after challenged by Ringer’s, the endocrine system and immune system could be regulated by the stress. The normal state in organism was broken, which caused the change of genes involved in these functions.

**TABLE 2:** DEGs associated with immune response in blood.

Gene	Annotation	*P*-Value
**BL-BC vs 8h**		
RBL	L-rhamnose-binding lectin CSL2-like	5.00E-05
MRC2	C-type mannose receptor 2	5.00E-05
SIG15	Sialic acid-binding Ig-like lectin 15	0.003
TLR5	Toll-like receptor 5	0.0001
PRS57	Serine protease 57	0.0076
CLC5A	C-type lectin domain family 5 member A	5.00E-05
TCB	T-cell receptor beta, type 1	5.00E-05
CCL2	C-C motif chemokine 2	0.01115
XLOC_009588	Galectin-3-like	0.0036
CD2	T-cell surface antigen CD2-like isoform X1	0.00245
NITR	Novel immune-type receptor	5.00E-05
IL21R	interleukin-21 receptor-like isoform X1	5.00E-05
HHLA2	HERV-H LTR-associating protein 2-like	5.00E-05
Nfkbid	NF-kappa-B inhibitor delta-like	0.01315
CD81	CD81 antigen	0.01425
**BL-BC vs 48h**		
TLR5	Toll-like receptor 5	5.00E-05
TNIP1	TNFAIP3-interacting protein 1-like	0.00525
MRC2	C-type mannose receptor 2	0.00215
IL17RB	Interleukin-17 receptor B	5.00E-05
TLR2	Toll-like receptor 2	5.00E-05
TLR1	Toll-like receptor 1	5.00E-05
CD9	CD9 antigen	0.0016
CLEC	C-lectin-A	0.0001
XLOC_002185	Immunoglobulin light chain 2	0.00345
NLRC3	NOD-like receptor C	5.00E-05
TNFRSF11	Tumor necrosis factor receptor superfamily member 11A	0.01615
TLR3	Toll-like receptor 3	0.0002
APOL6	Apolipoprotein L6	5.00E-05
XLOC_013403	Galactose-specific lectin nattectin	5.00E-05
IL21R	Interleukin-21 receptor	5.00E-05
APOL3	Apolipoprotein L6	5.00E-05
TLR7	Toll-like receptor 7	0.0013
CCL20	C-C motif chemokine 20	0.0102

**TABLE 3:** DEGs associated with immune response in gill.

Gene	Annotation	*P*-Value
**G-BC vs 8h**		
CLEC3A	C-type lectin domain family 3 member A	5.00E-05
CCL4	C-C motif chemokine 4	0.01045
SOX10	Transcription factor SOX-10	5.00E-05
IL34	Interleukin-34	0.0198
CCL20	C-C motif chemokine 20	0.0022
BCAP31	B-cell receptor-associated protein 31	5.00E-05
B2L12	Bcl-2-like protein 12	5.00E-05
TLR5	Toll-like receptor 5	5.00E-05
TNFRSF1A	Tumor necrosis factor receptor superfamily member 1A	0.0171
IL13R2	Interleukin-13 receptor subunit alpha-2	5.00E-05
FCGBP	IgGFc-binding protein	5.00E-05
HHLA2	HERV-H LTR-associating protein 2-like	5.00E-05
SOCS5	Suppressor of cytokine signaling 5	0.00405
CASP9	Caspase-9	5.00E-05
CD276	CD276 antigen	5.00E-05
**BL-BC vs 48h**		
CCL4	C-C motif chemokine 4	0.0022
LAG3	Lymphocyte activation gene 3 protein	5.00E-05
SOCS3	Suppressor of cytokine signaling 3	5.00E-05
CCL19	C-C motif chemokine 19	5.00E-05
RORA	Nuclear receptor ROR-alpha	0.0041
NPNT	Nephronectin	5.00E-05
NAL12	NACHT, LRR and PYD domains-containing protein 12	0.00265
CD276	CD276 antigen	5.00E-05
CCL20	C-C motif chemokine 20	0.0117

**TABLE 4:** DEGs associated with immune response in kidney.

Gene	Annotation	*P*-Value
**K-BC vs 8h**		
GLEC	Galactose-specific lectin nattectin-like	0.02125
TNIP1	TNFAIP3-interacting protein 1	0.0008
XLOC_012310	Immunoglobulin light chain 2	5.00E-05
TUSC2	Tumor suppressor candidate 2	0.00705
HSP60	Heat shock protein 60 kDa	5.00E-05
CD81	CD81 antigen	5.00E-05
IL21R	Interleukin-21 receptor	5.00E-05
CD34	Hematopoietic progenitor cell antigen CD34	0.01775
CD59	CD59 glycoprotein-like isoform X1	5.00E-05
VSIG1	V-set and immunoglobulin domain-containing protein 1	0.00035
**K-BC vs 48h**		
TLR13	Toll-like receptor 13	5.00E-05
IL11	Interleukin-11	0.0222
XLOC_012310	Immunoglobulin light chain 2	5.00E-05
BOKA	Bcl-2-related ovarian killer protein homolog A	5.00E-05
ISLR2	Immunoglobulin superfamily containing leucine-rich repeat protein 2	5.00E-05
IGSF8	Immunoglobulin superfamily member 8	5.00E-05
CD9	CD9 antigen	0.0038
IL8	Interleukin-8	5.00E-05
HSPB	Heat shock protein beta-1	5.00E-05
CD28	T-cell-specific surface glycoprotein CD28	5.00E-05
IGSF3	Immunoglobulin superfamily member 3	0.002
CD97	CD97 antigen	5.00E-05
CD109	CD109 antigen	5.00E-05
IGLL5	Immunoglobulin lambda-like polypeptide 5	5.00E-05
TLR9	Toll-like receptor 9	5.00E-05
IL17RB	Interleukin-17 receptor B	0.0009
TLR8	Toll-like receptor 8	5.00E-05
CD2	T-cell surface antigen CD2	5.00E-05
CD82	CD82 antigen	5.00E-05
CD267	CD276 antigen homolog	5.00E-05
TCB2	T-cell receptor beta, type 2	5.00E-05
SIK2	Serine/threonine-protein kinase SIK2	5.00E-05
TNIP1	TNFAIP3-interacting protein 1	0.00795
TLR5	Toll-like receptor 5	5.00E-05
NLRC	NOD-like receptor C	5.00E-05
IL5RA	Interleukin-5 receptor subunit alpha Interleukin-1 receptor	5.00E-05
IL1RAPL1B	Accessory protein-like 1-B-like isoform X4	0.00975
VSIG1	V-set and immunoglobulin domain-containing protein 1	0.00205
SERP1	Stress-associated endoplasmic reticulum protein 1	5.00E-05
TLX1	T-cell leukemia homeobox protein 1	5.00E-05

**FIGURE 4:**
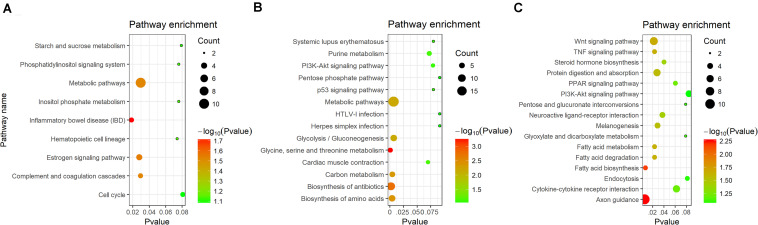
KEGG enrichment analysis of DEGs in different tissues. **(A)** KEGG enrichment in blood. **(B)** KEGG enrichment in gill. **(C)** KEGG enrichment in kidney.

KEGG pathway enrichment were analyzed in DEGs. A total of four signaling pathways were enriched significantly in blood (*P* < 0.05) (Figure 4A). In gill, six signaling pathways were detected (*P* < 0.05) (Figure 4B). In kidney, 10 signaling pathways were enriched significantly (*P* < 0.05) (Figure 4C). In these pathways, immune-related signaling pathways were significantly enriched, including inflammatory bowel disease, PI3K-Akt signaling pathway, p53 signaling pathway, and TNF signaling pathway. Interestingly, metabolic-related pathways were also found after challenge, such as metabolic pathways, carbon metabolism, glycolysis/gluconeogenesis, and fatty acid metabolism. The proteins inducing apoptosis and regulating inflammatory factors would be increased after stress challenge (Dios et al., 2007). It was necessary to supply energy by glycolysis or metabolism if apoptosis and inflammatory response were activated. We speculated that ATP was necessary to participate apoptosis and the phosphorylation of immune-related kinase when the stress of Ringer’s injection occurred. The process of immune response was accompanied with glycolysis or gluconeogenesis.

### Weighted Gene Co-expression Networks Analysis

**FIGURE 5:**
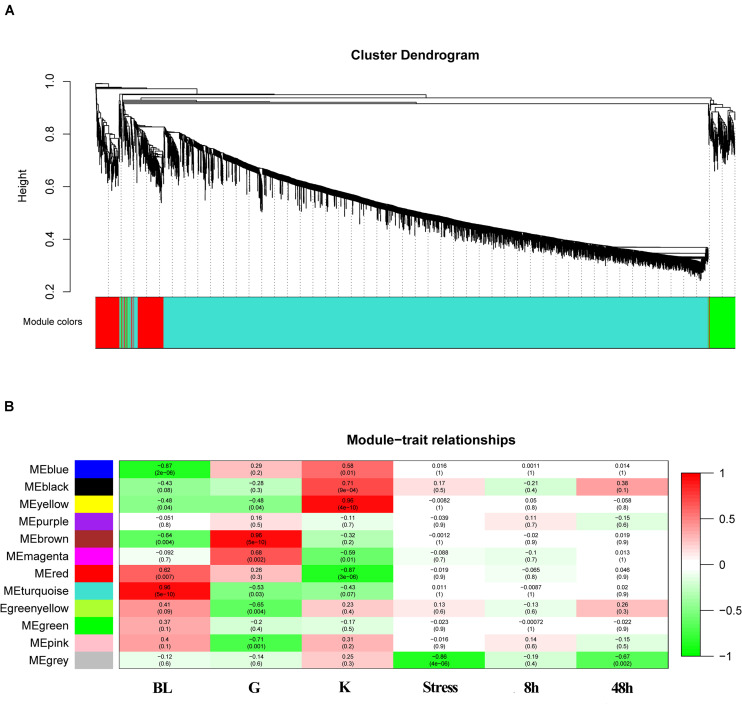
WGCNA analysis of the expressed genes. **(A)** Gene clustering tree (dendrogram) obtained by hierarchical clustering of adjacency-based dissimilarity. The colored row below the dendrogram indicates module membership identified by the dynamic tree cut method with module colors. **(B)** Module-feature associations. Each row corresponds to a module eigengene and each column to a trait. Each cell contains the corresponding correlation in the first line and the *P*-value in the second line. The table is color-coded by correlation according to the color legend.

Weighted gene co-expression networks analysis was constructed basing on all the genes expressed in three tissues. The correlation matrix and adjacency matrix of the gene expression profile of the challenged groups were calculated according to the basic idea of WGCNA. The challenged groups of 8 h and 48 h were delimited as stress groups. Then they were transformed into a topological overlap matrix (TOM) and obtained a system clustering tree of genes by gene-gene non-ω similarity (Figure 5A). The hierarchical average linkage clustering method was used to identify the gene modules of each gene network. A total of 12 gene modules were recognized in stress groups (Figure 5A). The highest association in the Module-trait relationship was found in gray module (*r*^2^ = 0.86, *P* = 4e-06) (Figure 5B). The ME gray module was selected as the module related to stress. In this module, 16 genes were chosen and were considered to be down-regulated stress (Table 5). The selected genes were immune-related and metabolic-related genes. It was consistent with the GO and KEGG analysis.

**TABLE 5:** Genes selected by WGCNA.

Gene	GS. Stress	*P*. GS. Stress
Retrotransposable element Tf2 155 kDa protein type 1	–0.873974332	2.14E-06
RNA-directed DNA polymerase from mobile element jockey	–0.867444433	3.14E-06
Zinc finger protein 45-like	0.839499762	1.32E-05
Unnamed protein product	0.78839998	0.000101502
Transposable element Tc1 transposase	–0.677349714	0.002013609
Myotubularin-related protein 7-like	–0.625467925	0.00550202
Probable RNA-directed DNA polymerase from transposon BS	–0.58350611	0.011018785
Interferon alpha-inducible protein 27	–0.539770239	0.02077184
NUAK family SNF1-like kinase 2-like	0.489045795	0.039434092
Bifunctional methylenetetrahydrofolate dehydrogenase/cyclohydrolase, mitochondrial-like	–0.477034255	0.045311857
Interferon alpha-inducible protein 27-like protein 2B like isoform X2	0.428551042	0.075992385
Ectonucleotide pyrophosphatase/phosphodiesterase family member 1	0.338264183	0.169757729
Uncharacterized protein	0.319036231	0.196904352
Neurexin-2-alpha-like	0.281091058	0.258508817
Interferon regulatory factor 3 variant 1	–0.189081435	0.452392985
Gamma-sarcoglycan-like	0.118463534	0.639662611

### qRT-PCR Validation

**FIGURE 6:**
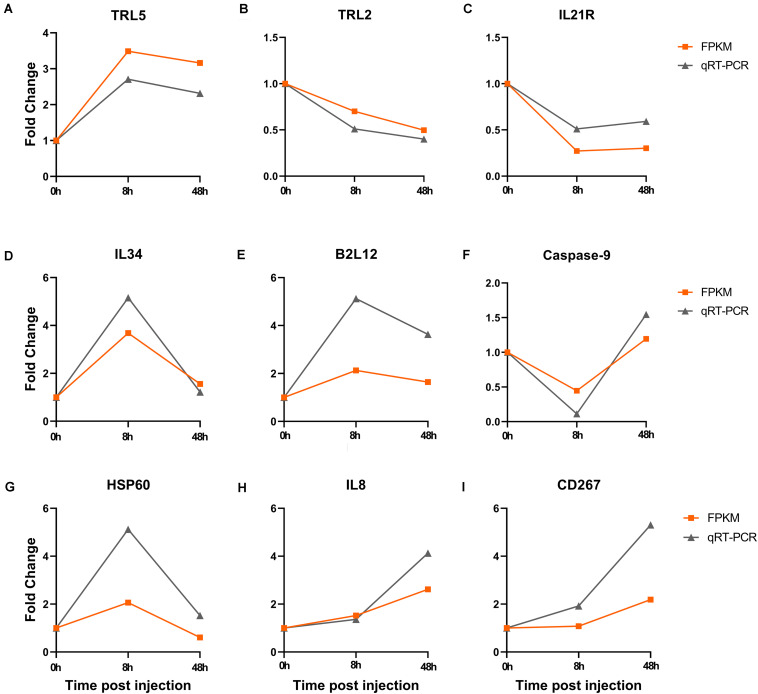
Verification of the fold change both in qRT-PCR and FPKM after Ringer’s injection. **(A–C)** The fold change of DEGs in blood at 8 h and 48 h post injection. **(D–F)** The fold change of DEGs in gill at 8 h and 48 h post injection. **(G–I)** The fold change of DEGs in kidney at 8 h and 48 h post injection.

The expression patterns of nine genes (TLR5, TLR2, IL21R, IL34, B2L12, Caspase-9, HSP60, IL8, CD267) related with immune response were detected by qRT-PCR. Fold change was calculated by treatment group and control group. All the genes displayed consistent expression patterns both in qRT-PCR and FPKM (*R* = 0.7613, *P* < 0.01) (Figure 6).

In conclusion, we demonstrated the change of expression profile post Ringer’s solution injection in Japanese flounder. The immune-related genes were up-regulated or down-regulated in different time points and different tissues. Besides, it was found that the genes involved in glycolysis/gluconeogenesis were also change after challenged with Ringer’s solution. These results indicated that the immune system and metabolic pathways were affected after Ringer’s solution challenge. This study revealed that Ringer’s solution buffer could influence immunostimulatory experiment during intraperitoneal injection.

## Data Availability Statement

The datasets presented in this study can be found in online repositories. The names of the repository/repositories and accession number(s) can be found in the article/Supplementary Material.

## Ethics Statement

The animal study was reviewed and approved by the Institutional Animal Care and Use Committee of the Ocean University of China.

## Author Contributions

JL and QZ conceived and designed the project, wrote and revised the manuscript. JL and ZL performed the experiments. JL and YW performed the data analysis. All authors read and approved the final manuscript.

## Conflict of Interest

The authors declare that the research was conducted in the absence of any commercial or financial relationships that could be construed as a potential conflict of interest.

## Supplementary Material

Supplementary Table 1 Primers used in this study.

Supplementary Table 2 DEGs between BC group and treatment groups at 8 h and 48 h post injection in blood.

Supplementary Table 3 DEGs between BC group and treatment groups at 8 h and 48 h post injection in gill.

Supplementary Table 4 DEGs between BC group and treatment groups at 8 h and 48 h post injection in kidney.
