# Supplementary material for: Impact of Ringer’s Solution Challenge Stress to Immunostimulatory Experiment, Insights From Japanese Flounder
Source: Front Physiol. 2020 Dec 4;11:612036. doi: 10.3389/fphys.2020.612036 (PMC7746848; doi:10.3389/fphys.2020.612036)
Supplement: Supplementary Table 1 — Primers used in this study. [file Table_1.docx]

**Table S1** Primer used in the study.

| Primers | Sequence (5′-3′) | Tm (°C) | Usage |
| --- | --- | --- | --- |
| TRL5-Fw  TRL5-Rv  TRL2-Fw | GAAGGCCTCTCGAATAGCGCTGTTA  GCACCCCTTGATAAAGTTGCTGCTG  CAATCAGCTGACGTCTCTGA | 60  60  60 | qRT-PCR  qRT-PCR  qRT-PCR |
| TRL2-Rv | GAAAAGGTTACGGAAGGTCA | 60 | qRT-PCR |
| IL21R-Fw | CACGCAGACTATTCCATTCA | 60 | qRT-PCR |
| IL21R -Rv | GCTCCACTCACTTCATACAC | 60 | qRT-PCR |
| IL34-Fw | GAGACAGAACATGTGGAAGAG | 60 | qRT-PCR |
| IL34-Rv | GTAGAACGTCTTGGGAGTAAAG | 60 | qRT-PCR |
| B2L12-Fw | CTCCTCCATCTCCTCAATCT | 60 | qRT-PCR |
| B2L12-Rv | TCTACCAGGCCTCTCTTTC | 60 | qRT-PCR |
| Caspase-9-Fw | CGTTTCCTGGTTACGTTTCT | 60 | qRT-PCR |
| Caspase-9-Rv | GGTCATCAGTAGCAGCATTT | 60 | qRT-PCR |
| HSP60-Fw | AGATACCCAAGGAGGAGAAG | 60 | qRT-PCR |
| HSP60-Rv  IL8-Fw  IL8-Rv  CD267-Fw  CD267-Rv | CGGGAGAGTCGGATTAGAA  TACCACTGACGGGTCAAAGT  TACTTTTGACTGGGGGTTCA  AGAGGGGTCTGTTTCACCTG  CTTGGGTTCCTAGGCTTGGT | 60  60  60  60  60 | qRT-PCR  qRT-PCR  qRT-PCR  qRT-PCR  qRT-PCR |
| β-actin-Fw | GCTGTGCTGTCCCTGTA | 60 | qRT-PCR |
| β-actin-Rv | GAGTAGCCACGCTCTGTC | 60 | qRT-PCR |
